# Discussion in Standing Committee—Second Reading

**Published:** 1919-04-19

**Authors:** 


### NURSES' REGISTRATION BILL.

### Discussion in Standing Committee—Second Reading.

The Standing Committee on the Bill sat for the second time on Thursday, April 10, 1919, in Committee Room 10, under the chairmanship of Mr. D. Macmaster, K.C. The Government amendments in the name of Dr. Addison were taken charge of by Sir Kingsley Wood, who explained that it was a matter of great difficulty for Dr. Addison to be personally present. It was thought at the last sitting, he explained, that the President of the Local Government Board should consider various clauses in this Bill, with the view of making some suggestions on behalf of the Government. Since that time the President had conferred with several members, and the scheme set out in the amendment paper had been arrived at. Every attempt had been made to give favourable consideration to all points of view. In setting down the amendments in his name the President had been animated from the public point of view in the first place, and, secondly, with the view of giving adequate representation to the numerous associations interested in this measure—bodies which had done such valuable work. He was sure neither the President nor any other member of the Committee desired to raise any question about this or that association; attempts had been made to give adequate representation to all. According to this scheme the number of the first-constituted Council was raised to thirty-one. One other person to be appointed by the Privy Council had been added. The Government had also adopted the suggestion put forward by the member for Lambeth (Mr. Briant), that none of these persons who should be appointed by the Privy Council shall be registered practitioners or nurses or persons engaged in, or associated with, the regular direction or provision of the service of nurses. Consideration had also been given to the claims of Wales, and one representative each would be accepted for the Principality under (*b*) and (*c*).

### Additional College Representation.

On Clause 4, Sub-section (1), par. (*k*), a suggestion had now been finally made for the Committee's consideration—namely, that there shall be eighteen women nurses, of whom eight shall be resident in England, two in Wales, four in Scotland, and four in Ireland, to be nominated subject to the approval of the Local Government Board for England, the Local Government Board for Scotland, and by the Local Government Board for Ireland respectively, by the following bodies and in the following numbers: one by the Queen Victoria's Jubilee Institute for Nurses; one by the Asylum Workers' Association; one by the North Wales and South Wales Nursing Associations—again giving additional representation to Wales—four by the Royal British Nurses' Association; additional representation in connection with the College of Nursing—on behalf of which body Lieut.-Colonel Raw had made strenuous efforts—namely, four; seven representing the following societies of trained nurses, by the Central Committee for the State Registration of Nurses: (1) The Matrons' Council of Great Britain and Ireland; (2) the Society for the State Registration of Trained Nurses; (3) the National Union of Trained Nurses; (4) the Fever Nurses' Association; (5) the Scottish Nurses' Association; (6) the Irish Nurses' Association; (7) the Irish Nursing Board. While members might be disposed to say some association should have more representation, or that some body was not represented at all, he hoped the Committee would regard what was now proposed as a fair compromise under the difficult circumstances, and that they would support the President in resisting, if necessary, any attempt to disturb this arrangement. He made an earnest appeal to the Committee on those lines. He moved to leave out "twenty-eight" in Clause 4 and substitute "thirty-one."

A member of the Committee made an appeal for direct representation of children's nurses on this Council. There were, he said, a large body of nurses who had done three years' work in children's hospitals and were fully qualified. He thought they should be given the same status as other nurses. He was not in a position to suggest a nomination.

Major Barnett replied that there was nothing in the Bill which would exclude children's nurses' representatives being nominated under Section (*e*).

### Societies with High-sounding Names.

Mr. Lyle, in the course of an able and convincing speech of considerable length, said he had submitted a manuscript amendment to Clause 4, substituting for twenty-eight, thirty-five. He realised he was taking a bold course. The promoters of the Bill, the representatives of the College of Nursing, and the Local Government Board, having sat round a conference table and, in their joint wisdom, agreed upon this Constitution, it was a bold thing for anybody to suggest it was an unsound one. Nevertheless, he felt it his duty to place his views before the Committee. He submitted that the Constitution suggested in the amendment of the President of the Local Government Board was entirely unsound. Mr. Lyle's reason for desiring to raise the number to thirty-five was that not a single place was given to any representatives of voluntary hospitals or to Poor-Law infirmaries. Whenever he had mentioned these he had been met by the remark that those bodies were employers, and employers could not be represented. He contended, however, that though we were now living in democratic days, surely we had not arrived at the time when employers should have no representation. Was it right and fair that the representatives of these big training-schools for nurses—who were elected by the governors and subscribers, and in the case of the Poor-Law infirmaries were directly elected by the people—should have no representation? In the Permanent Council the principle was recognised, because under Clause 4 (*f*) four persons were to be appointed by the nurse training-schools attached to hospitals approved by the Council. His suggestion was to give two seats to the voluntary hospitals and two to Poor-Law infirmary managers. Each had its particular organisation, and could easily nominate a representative. He congratulated Major Barnett on having succeeded in persuading the President of the Local Government Board to give these high-sounding but somewhat senile societies the same representation as was suggested before. When the President undertook to have a consultation on the competing claims of two big societies—he was not in any way connected with the College of Nursing—one would have thought he would have gone into the relative strength and status, their standing, power, and position. But apparently that had not been done. For instance, the Matrons' Council of Great Britain and Ireland carried a high-sounding name, but it was an unrepresentative body of the matrons of the country. He had been told on good authority that last year's receipts of that body amounted to £4 7s. 6d. The College of Nursing had only four representatives, yet its membership was 13,500 fully-trained nurses, and it could not be regarded as an employers' association. The representation suggested by the President was unfair and inequitable. His object was not to wreck the Bill, but he submitted that the absence of representation of voluntary hospitals would arouse much opposition from certain people who were opposed to the whole principle of the State registration of nurses.

Major Barnett admitted that Mr. Lyle did not get an opportunity of discussing the amendment as now drafted. The representative of the College of Nursing, Ltd., agreed to this compromise, and the present scheme had been arrived at after much discussion and give-and-take. The representation given to the College was the same as that for the Royal British Nurses' Association, the only nursing body with a Royal Charter. Arguing against the inclusion of the voluntary hospitals and the Poor-Law infirmaries, he

**Nurses' Registration Bill—(*continued*).**

reminded Mr. Lyle that there are 1,500 infirmaries in the kingdom, and to give them adequate representation would require a Franchise Act.

Lieut.-Colonel Raw pointed out that at the bottom of the efforts was the desire to avoid the measure becoming a controversial one; the wish was that the principle of registration should be carried out for the benefit of all nurses. But he thought the Poor-Law nurses, numbering 40,000 and treating two-thirds of the sick people of this country, should have at least one representative. The College of Nursing was not adequately represented in this scheme, but for the sake of harmony he agreed to the compromise.

Sir Kingsley Wood, in making a further appeal for unanimity, said he did not see why the Matrons' Council of the National Union of Trained Nurses should not put forward a representative of the voluntary hospitals.

Mr. Lyle's amendment was lost, and the Government amendment, increasing the number to thirty-one, was adopted.

Sir Kingsley Wood moved, and the Committee agreed, to increase the number of persons to be appointed by the Privy Council to four, and to add, after the foregoing, the words "not being registered medical practitioners, or nurses, or persons engaged in, or associated with, the regular direction or provision of the service of nurses"; also to provide that two medical practitioners shall be appointed by the Local Government Board for England (one for England and one for Wales). The Committee also agreed to four (instead of three) being appointed by the British Medical Association, one such medical practitioner to be resident in Wales.

The Committee then passed the revised Constitution, as described by Sir Kingsley Wood in his opening speech.

The Committee approved the addition by Major Barnett to Sub-section (2), Clause 4, which makes it read, "The members of the Council shall appoint one of the members of the Council to be President of the Council, and also one of the members of the Council to be Vice-President of the Council."

Fees for Attendance.

On the motion that the clause, as amended, do stand part of the Bill,

Commander Wedgwood asked whether the members of the Council were to be paid for their services, and, if so, was there a provision to pay them out of the registration fees. If they were not to be paid, would they receive an allowance for travelling expenses to attend Council meetings? If the meetings were to be held in London, it would be almost impossible for nurses to attend unless they had their railway fares paid.

Major Barnett pointed out that Clause 18 provides that members of the Council shall be paid such fees for attendance approved by the Privy Council and such reasonable travelling expenses as shall from time to time be allowed by the Council. These sums would come out of the Council's funds.

Clause 4 was then agreed to, also Clause 5.

Proportional Representation Introduced.

On Clause 6, Commander Wedgwood pleaded that this was a good opportunity for introducing the principle of proportional representation in elections, which was the only one to secure a true result. Its working was very simple, and the case would be met by altering the clause in accordance with the amendment put down in the name of the honourable member for Consett.

Several members of the Committee supported the amended form.

Major Barnett objected to the experiment being tried in this case, especially as "plumping" would not be allowed.

Major Barnett and Sir Kingsley Wood suggested that the matter should be left over until the report stage, when a workable plan might be produced, but the amendment was carried on a division by twelve votes to nine.

On Clause 11, Mr. Cowan's amendment of par. (*b*) was accepted by the promoters as follows: "Regulating the conditions of admission to the register of Nurses, the issue of certificates of registration, and the cancelling thereof."

The clause was accepted, subject to amendment of Clause 15 in accordance with it.

Unregistered Nurses.

Commander Wedgwood asked what action was proposed to be taken against nurses who continued to act as such without being on the Register. He understood there was not to be a colourable imitation of the uniform, and those persons were not to call themselves registered nurses. He wanted to know whether action was contemplated against the numberless middle-aged women who came in at times of sickness and charged very small fees, who were accustomed to wear the same kind of nurses' uniform, and to be called "nurse." He hoped it was understood there was to be no sudden burst of persecution of these people, the result of which would be to drive them entirely out of the profession. These people, who were usually in a poor position in life, should be allowed to continue in their work for a period of years.

Sir Kingsley Wood asked that the point be brought up on Clause 21. Clause 19 set out the penalties. The object of the Bill was to give a status to the nurses who were registered. The Council would fix the badge or uniform, and people not registered must not use it. The offence must be *knowingly* committed; and the penalty was a fine not exceeding £10.—Clause 11 was approved.

Major Barnett moved to leave out of Clause 12, Subsection (1), the words from "training" to "from," which makes the clause read: "(1) holds a certificate of training from a hospital or from hospitals approved by the Council, or from an institution or institutions which the Local Government Board recommend and certify to be wholly or partly maintained out of rates." It was to ensure that people carrying on the *bona-fide* business of a nurse should get on to the Register. The amendment was agreed.

On Clause 12, Sub-section (4), Sir Kingsley Wood, on behalf of the Government, moved to leave out from "of" to the end of the sub-section (as to training), and insert "having been for at least three years in *bona-fide* practice as a nurse in attendance upon the sick, and as to the conditions under which she was so engaged." He said it was an important amendment. Sub-clause 2 provided that nurses must hold a certificate of training from the Admiralty for the sick berth staff of the Royal Navy, or as a nurse authorised by the Army Council for soldiers of the Royal Army Medical Corps, or from the Local Government Board for Ireland, or the similar Board for Scotland, and had been at least three years, in addition, in *bona fide* practice as a nurse. As originally drafted, it was in very wide terms, and the Council might be under all sorts of pressure. As now framed, it would rule out nurserymaids and other people who would not make an attempt to get on to the Register: the Council must be satisfied as to the conditions under which the nurse had been engaged.

Mr. Lyle asked the meaning of the words "*bona-fide* practice."

Sir Kingsley Wood replied that there had been many cases in connection with dentists and medical practitioners in which those words had been discussed, and they carried a well-known and understood meaning. Roughly, it meant that the person must have been a real nurse in proper practice. It was a question of fact which, no doubt, the Council would be able to decide. In further reply to Mr. Lyle, he said the three years' training was for future candidates. The clause, as amended, was agreed to.

The further deliberations of the Committee were adjourned.

---

The Bill was further considered in Committee on Monday afternoon.

**Nurses' Registration Bill**—(*continued*).

The Right of Appeal.

Lieut.-Colonel Raw moved to leave out Clause 14, which says: "If the Council refuse to recognise any hospital or institution as an approved training school for nurses under this Act, the governing body of such hospital or institution or any person aggrieved by such refusal may make a representation to the Privy Council, and the decision of the Privy Council shall be binding on the Council." He observed that it was a matter of great importance to the nursing profession, and there seemed to be considerable difference of opinion as to whether or not it would be wise for nurses to have a Court of Appeal at all. The members of the medical profession, on matters of conduct, had no appeal beyond the General Medical Council, but the responsibility connected with this particular clause was so great that he preferred that the Committee as a whole should share it. The new Ministry of Health would take over some of the powers now exercised by the Privy Council, and he asked Sir Kingsley Wood to state, on behalf of the Government, whether the Ministry of Health being substituted for the Privy Council would receive support.

Sir Kingsley Wood replied that he thought some right of appeal should be retained. It was true that the Ministry of Health would have power to take over certain health functions which at present appertain to the Privy Council, and he thought he could say that this subject would receive Dr. Addison's consideration. Meantime he urged that there should be a right of appeal, and that at present, at any rate, the body was, rightly, the Privy Council.

Clause 14 was approved; also Clause 15.

The Children's Nurses' Register.

On Clause 16, Mr. Lyle moved to add: "(4) A supplementary register of children's nurses, to be called the Children's Nurses' Register, containing the names of children's nurses who have been registered under this Act." He said he understood Major Barnett had agreed to accept these or similar words. He thought that nurses who had taken over the responsibility of nursing children and wished to nurse children all their lives should be given some protection and a status in the nursing profession. There was no phase of nursing which was better or more needed at the present time; the best type of woman was required for it, and she should have protection. He was not asking that these particular women should be called fully trained nurses, and the nurses concerned did not themselves ask for it. They merely asked that there should be a supplementary register which should contain their names and particulars. In the Bill as at present drafted such nurses were not singled out as against the persons who masqueraded in nurses' uniform. Children's nurses should have the same status as mental nurses and the other classes set out in the Bill.

Colonel Burn strongly supported the plea.

Commander Wedgwood said the object was to establish a caste system in the profession; nobody wanted to include those who wheeled a child about in a "pram" in the park.

After further debate, Mr. Lyle agreed to accept Col. Raw's amendment in place of his own, and it was by leave withdrawn.

Lieut.-Colonel Raw moved to add "(4) A supplementary Register of nurses trained in nursing sick children, to be called the 'Children Nurses' Register,' containing the names of children's nurses who have been registered under this Act." He explained that this referred only to nurses who had undergone a three-years' period of training in a recognised children's hospital, such as that in Great Ormond Street, London, and at Pendlebury, Manchester, and who intended to devote their lives to nursing sick children. He was not in favour of Supplementary Registers generally, but thought a good case was made out for children's nurses. Should this amendment not be carried the effect would be that many hospitals for sick children would not be able to get nurses to carry on their good work, because one could not expect nurses to devote themselves to that special work if they were not allowed to go on to a Register. In reply to a question, he said these children's nurses would have the right to nurse adult persons if they wished.

The amendment was agreed to.

A Fever Nurses' Register?

Sir Watson Cheyne wished to put in a plea that nurses who had received training in fever hospitals should be available by the public for cases of fever nursed in their homes. In Scotland, in the last three years, 33,700 fever patients were nursed by the 5,000 fever nurses. He asked that there should be a Supplementary Register for these. He moved to add for Clause 16, line 28, "A Supplementary Register of fever nurses, to be called the 'Fever Nurses' Register,' containing the names of nurses who have been trained for three years at a fever hospital recognised by the Council, and who hold the certificate of the Fever Nurses' Association or its equivalent under conditions approved by the Council."

Sir Kingsley Wood, in consequence of the shortness of notice, asked the Committee to agree to defer its decision on this and give the Government an opportunity of conferring with those interested in the matter.

On this understanding the amendment was, by leave, withdrawn.

The Case of the Village Nurse.

Sir Watson Cheyne also, without making a motion, drew attention to the case of the village nurse, who was a very useful and important person in small and out-of-the-way communities. Many rural districts—and he was thinking especially of Scotland—could not afford a fully-trained nurse, and women rose up in those districts and performed most valuable ministrations to the poor people. He thought an endeavour should be made to organise those nurses, and enable them to get a certain amount of training. Many would be willing to leave their village for a time to secure this, returning to bring to bear their better methods so acquired.

Clause 16, as amended, was agreed to.

The Fees for Registration.

On Clause 17, Sir Kingsley Wood moved, on behalf of the Government, to insert at the end "such fee not to exceed the sum of one guinea for registration under Section 12 of this Act, or three guineas for examination and registration under the remaining provisions of this Act." He said that at present the Bill left the question of fee to the Council, and that was not thought to be a desirable course.

Major Barnett urged that this amendment was somewhat drastic from the point of view of finance. If its acceptance would conciliate some of the opposition which had been shown to the Bill he would agree to it, otherwise he would ask that there be a reversion to the original proposition. He asked the Government to accept the addition of the words: "Such fee not to exceed the sum of one guinea for registration within twelve months after the passing of this Act, or two guineas thereafter, or three guineas to the examination and registration under Section 13 of this Act." The object, he said, was to induce nurses to register in the first year; it would be good for the finances of the scheme. When the Midwives Act was passed there was a reduction of fee for the first six months.

Sir Kingsley Wood agreed to accept that on behalf of Dr. Addison, and Lieut.-Colonel Raw said it seemed to be a very reasonable compromise.

On a division it was carried by 12 to 11.

An Annual Fee.

Major Barnett moved "(2) There shall also be payable on or before the thirty-first day of January in each year by every registered nurse a fee of two shillings and sixpence, and if any nurse in any year makes default in paying such fee his or her name may be removed from the Register, but may be restored on payment of a fee not exceeding five

**Nurses' Registration Bill—(continued).**

shillings, and on proof that the failure was due to inadvertence or mistake, or on giving other satisfactory explanation." He said this restored a provision which was originally in the Bill. It would keep the Register fresh, and would be no hardship.

A Member said it would have the added advantage that the Register would then embrace only the names of those actively engaged in practice, and would thus be a dependable, up-to-date record. Half-a-crown would not be a serious burden to any nurse.

It was urged by another member that, because a nurse paid a few months late she should not be required to pay five shillings instead of half-a-crown. Also that sixpence would cover the expense incidental to the registration. An amendment on these lines was pressed to a division, and was carried by one vote—twelve to eleven. Following upon that, the fee for restoration to the Register was reduced from five shillings to one shilling, Major Barnett stating that the alteration meant a reduction of £6,000 a year.

Major Farquharson, before the alteration was agreed to, declared that though nurses were admittedly the "worst-paid set of people on earth," nurses themselves would welcome some method of keeping before the public the clear-cut quality of this Register as an agent for the public good, quite apart from this question of weighing sixpences; and they would not mind paying for it. To leave the fee at sixpence was a mere travesty of the position; nurses' names would remain on the Register years after they were dead. He hoped this would be remedied on reaching the report stage of the Bill.

The Status of the Auditor.

Mr. Wm. Graham moved to leave out from Clause 17 the words, "who shall be a member either of the Institute of Chartered Accountants or of the Incorporated Society of Accountants, or of the Central Association of Accountants," and insert "has been admitted as a member of an incorporated society of accountants." He desired that there should be a wider field; for instance, nurses might desire a woman accountant. Moreover, he thought it was undesirable to leave it to the approval of the Board of Trade, as provided in Sir Samuel Scott's amendment. The Board would incline to approve the older societies rather than the more up-to-date ones.

It was agreed that this should be discussed with the amendment of which Sir Samuel Scott had given notice—namely, to leave out the words "or of the London Association of Accountants, or of the Central Association of Accountants," and to insert "or accountant or auditor approved by the Board of Trade." In furtherance of this amendment, Sir Samuel Scott said it was based upon a model clause which, in about the year 1914, was agreed upon by the Chairman of Committees of the House of Lords and the Chairman of Committees of the House of Commons to be inserted into all private Bills. After discussion the matter went to a division, when Mr. Graham's amendment was lost by eleven to ten, and Sir Samuel Scott's was agreed to.

Clause 17, as amended, was then approved; also the remaining clauses, and the Bill, as amended, was passed.

An Official Notice.

We are requested to publish the following special notice:

The Council of the College of Nursing desires emphatically to state that the amendment proposing a Children's Nurses' Register was not moved by Colonel Raw as the representative of the College of Nursing. On the contrary, the Council has always maintained that the question of supplementary registers should be left to the decision of the nurses who, under the College Bill, would have been in the majority to the extent of two-thirds on the General Nursing Council.

